# Does the impact of metabolic syndrome on cardiovascular events vary by using different definitions?

**DOI:** 10.1186/s12889-015-2623-3

**Published:** 2015-12-29

**Authors:** Hossein Khosravi-Boroujeni, Faruk Ahmed, Masoumeh Sadeghi, Hamidreza Roohafza, Mohammad Talaei, Minoo Dianatkhah, Ali Pourmogaddas, Nizal Sarrafzadegan

**Affiliations:** Menzies Health Institute Queensland, and School of Medicine, Griffith University, Gold Coast Campus, QLD Australia; Isfahan Cardiac Rehabilitation Research Center, Cardiovascular Research Institue, Isfahan University of Medical Sciences, Isfahan, Iran; Saw Swee Hock School of Public Health, National University of Singapore, Singapore, Singapore; Isfahan Cardiovascular Research Center, Isfahan University of Medical Sciences, Isfahan, Iran

**Keywords:** Metabolic syndrome, Obesity, Diabetes, Hypertension, Cardiovascular diseases

## Abstract

**Background:**

Metabolic Syndrome (MetS) is a complex disorder which increases the risk of chronic diseases, including cardiovascular diseases and diabetes mellitus. As a result of modern lifestyles, the prevalence of MetS has been rising globally. This study aims to investigate whether overall prevalence of MetS varies when using different definitions of MetS and to identify the best and most predictive definition of the MetS for cardiovascular disease (CVD) events over 10 years in a cohort of an Iranian population.

**Method:**

Adults aged ≥ 35 years from urban and rural regions in central Iran were selected at baseline and followed up for more than 10 years. Data on socio-demographic characteristics, anthropometry, blood pressure and smoking status were collected at baseline. In addition, various biochemical indices were assessed. MetS was defined based on five available definitions, and cardiovascular events during 10 years follow up were confirmed by an expert group. The hazard ratios were calculated by the Cox proportional hazards model.

**Results:**

The highest prevalence of MetS was observed by using AHA-NHBI definition (36.9 %), followed by JIS definition (31.2 %). On the other hand, EGIR (8.8 %) provided the lowest prevalence. The risk of developing CVD, irrespective of definitions, was approximately two fold higher in the presence of MetS. After controlling for possible confounders, AHA-NHBI definition was found to be the best predictor of CVD.

**Conclusion:**

This study demonstrated a great variability in the prevalence of MetS among Iranian adults when using different definitions of MetS. CVD risk was significantly higher in MetS participants, as well as in participants with any risk factors of MetS; however, the AHA-NHBI definition was found to be the best predictor of CVD. Thus protective measures, including lifestyle modifications, plus control of individual risk factors is necessary to prevent cardiovascular events.

## Background

Metabolic Syndrome (MetS) is a complex disorder with a collection of related metabolic risk factors which increase the risk of developing chronic diseases, such as atherosclerotic cardiovascular disease (ASCVD) and diabetes mellitus [1]. MetS is also associated with other disorders such as fatty liver [2], cholesterol gallstones [3], polycystic ovary syndrome [4], and sleep apnea [5]. In addition, it poses a significant risk of higher morbidity, mortality and financial burden [6]. The prevalence of MetS has been rising in both developed and developing countries, probably as a consequence of modern lifestyle and the overweight/obesity epidemic [7]. Therefore, MetS is considered a public health, as well as a clinical, problem. During the past decades, due to major lifestyle changes and aging population, the prevalence of MetS, cardiovascular diseases (CVD) and other chronic diseases has been increasing in Iran [8]. Based on a national study, MetS has been diagnosed in 34.7 to 37.4 % of the Iranian population [9]. Moreover, high incidence rates for almost all CVD and mortality have been reported in the Iranian population [10].

The etiology of MetS has not been clearly defined, thus the definition of MetS is not based on etiology and pathology, but on the predictors of CVD as a primary outcome of MetS. It’s diagnostic criteria have been developed on the basis of best clustering of interrelated risk factors of CVD which occur simultaneously and can predict CVD events [11]. In the past, several expert groups have attempted to develop practical diagnostic criteria to characterize individuals who are at high risk of CVD. They included underlying and metabolic risk factors, characterized by insulin resistance or impaired blood glucose, central obesity, dyslipidemia (increase in triglycerides and decrease in high density lipoprotein cholesterol (HDL) levels) and hypertension. However, the suggested criteria varies to some extent and some individuals might diagnose with one or two definitions but not with others [12–14].

Because there is no exclusive definition for the diagnosis of MetS, its prevalence, incidence and its association with an increased risk of cardiovascular diseases depends on the criteria used [15]. Thus, this study aims to investigate whether the prevalence of MetS varies when using different definitions of MetS in an Iranian population. In addition, it aims to determine the definition that is the best predictor for CVD events over 10 years in a cohort of an Iranian population.

## Methods

### Study design

The Isfahan Cohort Study (ICS) is an ongoing, population based, longitudinal study of adults aged ≥ 35 years, from urban and rural regions in central Iran. It is designed to display the incidence of CVD and its risk factors, and to determine the Iranian risk assessment values. Participants were selected between January and September 2001 by multistage random sampling and were enrolled to represent the age, gender and urban/rural distribution of their societies. The study details are presented elsewhere [16]. The study was approved by the Ethics Committee of the Isfahan Cardiovascular Research institute (ICRI) a World Health Organization (WHO) collaborating center and the Griffith University Ethics Committee.

### Measurements

After obtaining the informed written consent of participants, physical examinations, fasting blood samples, and anthropometric measurements were carried out. Serum triglycerides, fasting blood glucose (FBG), and total cholesterol (TC), were determined using the enzymatic method [17]. Serum HDL-C was measured after precipitation of low density lipoprotein (LDL) and very low-density lipoprotein (VLDL) [18]. The LDL level was calculated by Friedewald formula [19]. Weight and height were measured by a calibrated scale and stadiometer with participants wearing light clothes and no shoes. Waist circumference (WC) was measured with non-elastic measuring tape at or below the costal margin (minimal waist) without compressing the tissue. Blood pressure was taken twice at 5 min interval in a sitting position with a mercury sphygmomanometer with an appropriate cuff for adults. The mean value of the two measurements was calculated and applied.

### Metabolic syndrome definitions

Among the available definitions for MetS, this study selected the most widely practiced definitions which were developed by various international expert groups and organizations. The MetS was defined according to five definitions (Table 1). Based on the WHO definition, insulin resistance is required for diagnosing MetS, along with two other risk factors among central obesity, high triglyceride, low HDL or hypertension [12]. The European Group for Study of Insulin Resistance (EGIR) defined MetS only for non-diabetic people [20]. The National Cholesterol Education Program, Third Adult Treatment Panel (NCEP ATP III) did not emphasise any risk factors, but the presence of any 3 of the 5 risk factors would qualify a person for MetS [14]. Based on the International Diabetes Foundation (IDF), abdominal obesity is a requirement in MetS definition, and having central obesity plus any other two risk factors are required for the diagnosis of MetS [13]. This definition insists on easy-to-use measures in clinical practice, and moreover, emphasises ethnic differences in recognising the cut-off point of abdominal obesity [21]. The American Heart Association (AHA) and the National Heart, Lung, and Blood Institute (NHLBI) accepted the ATP III definition, but reduced the threshold for impaired glucose tolerance (IFG) from 110 to 100 mg/dl [22]. A harmonized definition of MetS (a Joint Interim Statement (JIS)) formulated by several organizations including IDF, NHLBI, AHA, the World Heart Federation, the International Atherosclerosis Society and the International Association for the Study of Obesity attempted to develop a unified criteria for defining MetS. They agreed that a single cut-off point for WC is not suitable and should not be a required component. Furthermore, any 3 out of 5 components are adequate for MetS diagnosis [11].

**Table 1:** Different definitions of metabolic syndrome

	WHO	EGIR	NCEP ATP III	AHA	IDF	JIS
Definitions	insulin resistance together with two or more of the following:	Insulin resistance or impaired fasting glucose (IFG) plus two of the following:	Three or more of the following five risk factors:	Three or more of the following five risk factors:	Central obesity plus 2 other features	Three or more of the following five risk factors:
Fasting plasma glucose	__	≥ 6.1 mmol/l (110 mg/dl) but non-diabetic	≥ 6.1 mmol/l (110 mg/dl)	≥ 5.6 mmol/l (100 mg/dl)	≥ 5.6 mmol/l (100 mg/dl) or diagnosed type 2 diabetes	≥ 5.6 mmol/l (100 mg/dl)
Central obesity	Men: waist-hip ratio > 0.90	Men: waist circumference ≥ 94 cm	Men: waist circumference > 102 cm	Men: waist circumference > 102 cm	Men: waist circumference ≥ 94 cm,	Ethnic cut point for waist circumference^a^
	Women: waist-hip ratio > 0.85 and/or BMI > 30 kg/m2	Women: waist circumference ≥ 80 cm	Women: waist circumference > 88 cm	Women: waist circumference > 88 cm	Women: waist circumference ≥ 80 cm^a^ or BMI > 30 kg/m^2^	
Blood pressure	≥ 140/90 mmHg	≥ 140/90 mmHg or treatment	≥ 130/85 mmHg	≥130/85 mmHg	≥ 130/85 mmHg or treatment	≥ 130/85 mmHg
Triglycerides	≥ 1.7 mmol/l (150 mg/dl)	> 2.0 mmol/l (178 mg/dl)	≥1.7 mmol/l (150 mg/dl) or treatment	≥1.7 mmol/l (150 mg/dl) or treatment	≥1.7 mmol/l (150 mg/dl) or treatment	≥ 1.7 mmol/l (150 mg/dl) or treatment
HDL-cholesterol	Men: < 0.9 mmol/l (35 mg/dl)	< 1.0 mmol/l (39 mg/dl) or treatment	Men: < 1.03 mmol/l (40 mg/dl)	Men: < 1.03 mmol/l (40 mg/dl)	Men: <1.0 mmol/l (39 mg/dl)	Men: < 1.03 mmol/l (40 mg/dl)
	Women: < 1.0 mmol/l (39 mg/ dl)		Women: < 1.29 mmol/l (50 mg/ dl)	Women: < 1.29 mmol/l (50 mg/dl)	Women: <1.3 mmol/l (40 mg/dl) or treatment	Women: < 1.29 mmol/l (50 mg/dl)

^a^based on Iranian cut off point, Waist Circumference ≥ 95 for both sexes

### Follow up

With the purpose of verifying CVD events, the follow-up of participants was conducted using telephone call interviews and home visits when required, every two years. The participants were asked about their hospital admissions or any cardiac or neurological symptoms that led to visiting a physician. In 2007 and 2013, interviews, physical examinations and laboratory tests were repeated for all participants. The measurement methods were similar to the 2001 survey. Acute myocardial infarction (AMI), unstable angina (UA), and sudden cardiac death were considered as the indicators of ischemic heart disease. A panel of three cardiologists and one neurologist, unaware of the data related to risk factors, examined all the documents to confirm the CVD cases. For the purpose of this study, the data on CVD event were recorded up to the 2013 survey.

### Statistical methods

The prevalence of MetS was calculated by using different definitions in the total samples and also by considering sex, age category, region, education level, and occupation using SPSS crosstab. For univariate analysis, the data were compared between groups by the Student *t*-test or chi-squared analysis. Based on our variables (the follow up duration and the CVD event), the Cox proportional hazards model was chosen as the best multivariate approach for analysing survival time data to investigate the association of different definitions of MetS and its components with cardiovascular events. In the first model, the analysis was conducted using a crude model, and in the second model, age, sex, smoking status and physical activity were adjusted to remove the effects of covariates. For all analyses, statistical significance was considered at a level of 0.05. All data were analysed by using Statistical Package for the Social Sciences (IBM SPSS Version 22).

## Results

Overall, 3336 females and 3168 males participated in the first phase of the ICS. When compared between various definitions of MetS, the highest prevalence was observed using the AHA-NHBI definition (36.9 %), followed by JIS (31.2 %) and ATP III (30.0 %). On the other hand WHO (13.3 %) and EGIR (8.8 %) provided a much lower prevalence (Table 2). Considering all definitions, the overall prevalence of MetS was higher in females than in males. Using WHO and EGIR definitions, the prevalence of MetS rose with increasing age, while it increased only until 65–75y using all other definitions.

**Table 2:** Prevalence of metabolic syndrome by sex and age groups based on having MetS using different definitions

MetS definition						
	WHO	EGIR	ATPIII	AHA-NHBI	IDF^a^	JIS
Total	13.3 %	8.8 %	30.0 %	36.9 %	28.0 %	31.2 %
Sex						
Male	10.6 %	6.9 %	20.7 %	21.4 %	25.5 %	29.1 %
Female	15.9 %	10.7 %	38.8 %	51.7 %	30.2 %	33.2 %
Age Categories						
35–45	7.6 %	5.8 %	19.9 %	28.4 %	19.0 %	21.2 %
45–55	13.1 %	8.5 %	32.7 %	40.6 %	31.4 %	34.2 %
55–65	19.8 %	12.7 %	40.1 %	47.4 %	37.0 %	42.2 %
65–75	21.3 %	13.0 %	43.4 %	46.9 %	37.9 %	42.3 %
> 75	21.9 %	14.4 %	30.5 %	33.3 %	27.8 %	33.7 %

^a^Waist Circumference ≥ 95 for both sexes

Table 3 shows the presence of CVD events based on the development of MetS using different definitions. Irrespective of the definitions used, MetS was significantly associated with CVD events. Higher values of CVD risk factors (e.g. age, cholesterol, blood glucose and smoking) were also observed in individuals with CVD events. However, HDL-C was an exception in that there was no significant difference in the levels between the CVD events groups.

**Table 3:** Present of cardiovascular events based on different definition of metabolic syndrome

		Cardiovascular event		
		No	Yes	*P*
Presence of MetS by different definitions	MetS (N)	N (%)	N (%)	
WHO definition	Yes (766)	568 (11.8)	198 (25.5)	< 0.001
	No (4818)	4241 (88.2)	577 (74.5)	
EGIR definition	Yes (507)	386 (8.0)	121 (15.6)	< 0.001
	No (5077)	4423 (92.0)	654 (84.4)	
ATPIII definition	Yes (1701)	1337 (27.8)	364 (47.0)	< 0.001
	No (3883)	3472 (72.2)	411 (53.0)	
AHA-NHBI definition	Yes (2093)	1681 (35.0)	412 (53.2)	< 0.001
	No (3491)	3128 (65.0)	363 (46.8)	
IDF^a^ definition	Yes (1595)	1266 (26.3)	329 (42.5)	< 0.001
	No (3989)	3543 (73.7)	446 (57.5)	
JIS definition	Yes (1773)	1397 (29.0)	376 (48.5)	< 0.001
	No (3811)	3412 (71.0)	399 (51.5)	
Cardiovascular risk factors				
Age (years)		49.8 ± 11.3	58.0 ± 11.6	< 0.001
FBS (mg/dl)		87.0 ± 29.9	101.1 ± 48.5	< 0.001
Total Cholesterol (mg/dl)		212.2 ± 51.5	228.8 ± 55.8	< 0.001
HDL-C (mg/dl)		46.9 ± 10.4	47.0 ± 10.6	0.87
LDL-C (mg/dl)		127.6 ± 42.9	138.4 ± 46.3	< 0.001
Triglyceride (mg/dl)		188.2 ± 101.7	217.4 ± 114.5	< 0.001
Waist circumference (cm)		94.4 ± 12.2	97.4 ± 12.4	< 0.001
BMI		26.6 ± 4.4	27.2 ± 4.7	< 0.001
Systolic BP (mmHg)		120.1 ± 20.0	133.5 ± 24.2	< 0.001
Diastolic BP (mmHg)		77.7 ± 11.2	83.1 ± 12.8	< 0.001
Daily Physical Activity (Mets/h		882.4 ± 544.9	755.1 ± 562.9	< 0.001
Smoking				< 0.05
Current smoker		769 (16.0)	139 (18.0)	
Past smoker		270 (5.6)	64 (8.3)	
Never smoker		3763 (78.4)	570 (73.7)	

Data presented as number (percent) or mean ± Standard deviation

^a^Waist Circumference ≥ 95 for both sexes

The risk of developing CVD, considering all definitions, was approximately two fold higher in the presence of MetS (Table 4). As shown in the crude model, the MetS using the WHO definition predicted the highest risk for CVD followed by the JIS definition (HR: 2.41, 95 % CI: 2.05–2.83 and HR: 2.14, 95 % CI: 1.86–2.46 respectively). After controlling for possible confounders including age, sex, smoking status and physical activity, the risk of CVD decreased slightly and using the AHA-NHBI definition was found to be a better predictor than using other definitions (HR: 1.93, 95 % CI: 1.66–2.25). When examining the risk of CVD events for each of the abnormal components of MetS, the risk of CVD occurrence was also significantly higher. Among the components, glucose abnormality was found to be a higher predictor of CVD events (HR: 1.83, 95 % CI: 1.56–2.15) than the other components.

**Table 4:** Hazard Ratio of CVD occurrence based on different definitions of metabolic syndrome and its components

Metabolic syndrome definitions	Crude HR (95 % CI)	*P*-Value	Adjusted HR^b^ (95 % CI)	*P*-Value
WHO	2.41 (2.05–2.83)	< 0.001	1.92 (1.62–2.26)	< 0.001
EGIR	2.03 (1.67–2.47)	< 0.001	1.66 (1.37–2.03)	< 0.001
ATPIII	2.12 (1.84–2.44)	< 0.001	1.87 (1.61–2.16)	< 0.001
AHA-NHBI	1.98 (1.72–2.28)	< 0.001	1.93 (1.66–2.25)	< 0.001
IDF^a^	1.93 (1.67–2.23)	< 0.001	1.65 (1.43–1.91)	< 0.001
JIS	2.14 (1.86–2.46)	< 0.001	1.80 (1.56–2.08)	< 0.001
Metabolic syndrome components				
Obesity	1.51 (1.31–1.75)	< 0.001	1.44 (1.24–1.67)	< 0.001
High triglyceride	1.67 (1.43–1.95)	< 0.001	1.60 (1.37–1.87)	< 0.001
High LDL-C	1.49 (1.28–1.74)	< 0.001	1.30 (1.11–1.52)	< 0.01
Low HDL-C	1.07 (0.91–1.25)	0.404	1.19 (1.01–1.39)	< 0.05
Hypertension	2.51 (2.18–2.89)	< 0.001	1.79 (1.53–2.08)	< 0.001
Glucose intolerance/ Diabetes	2.21 (1.88–2.59)	< 0.001	1.83 (1.56–2.15)	< 0.001

^a^Waist Circumference ≥ 95 for both sexes

^b^Adjusted Model for age, sex, smoking status and physical activity

## Discussion

The present study demonstrated that the prevalence of MetS among Iranian adults varies widely when different definitions are used. Using the WHO and EGIR definitions resulted in a much lower prevalence of MetS when compared with other definitions. Regardless of the definitions, this study also revealed that diagnosing MetS can help identify individuals who are at a higher risk of CVD and can also predict long term CVD events. The AHA-NHBI definition was found to be the best predictor of CVD followed by the WHO and ATPIII definitions; nevertheless, the hazard risk ratios for all definitions were very close.

Researchers have found that multiple endogenous origin risk factors of CVD may accumulate in one person [23]. Thus, MetS has been defined by expert groups as a functional and simple indicator of the risk of CVD, although the predicted risk depends on which definition of MetS is used [23]. Some definitions have emphasised insulin resistance as an essential component for the diagnosis of MetS. For instance, according to the WHO definition, without insulin resistance, individuals would not have MetS, even though they have all other criteria [12]. Insulin resistance influences hyperglycemia and diabetes mellitus [24], and increases lipolysis of stored lipids and free fatty acids [25]. Furthermore, it can lead to vasoconstriction and sodium retention which ultimately cause hypertension [26]. Later, abdominal obesity was detected to be strongly associated with insulin resistance [27], impaired glucose tolerance (IGT) [28], hypertension [29], hyperlipidemia [30] and increased risk of coronary heart disease [31]. Abdominal obesity is metabolically active and releases bioactive products such as free fatty acids [32], inflammatory cytokines and adipokines [33]. Thus, abdominal obesity is implicated as a MetS risk factor, and consequently IDF has considered this kind of obesity to be an essential determinant of the MetS [27].

Surprisingly, it was confirmed that individuals having inherent insulin resistance, such as individuals with South Asian ethnicity, can develop insulin resistance and MetS without an excessive degree of obesity, and even with a WC below the cut off points [34]. In Asian populations the NCEP and ATP III definitions underestimated the population at risk [35]. Due to the ethnic differences, there has been a proposal for using modified cut-off points for defining central obesity as a risk factor for MetS [13]. In the current study, the Iranian National Committee of Obesity cut-off point for WC (≥ 90 cm for both genders) was used to determine the risk for CVD [36]. Further, it was suggested that there should not be any compulsory components [11].

Previous studies among different population groups in Iran have shown varied prevalence of MetS based on different definitions. However, the patterns of MetS prevalence, using different definitions [9, 37], were very similar to those observed in our study. For instance, in Zabetian et al’s study [37], the prevalence of MetS was 32.1, 33.2 and 18.4 % based on the IDF, ATP III, and WHO definitions respectively. In Delavari et al’s study [9] its prevalence was 37.4, 34.7 and 41.6 % based on the IDF, ATP III, and AHA/NHLBI criteria respectively. Other studies have also reported that the prevalence of MetS was approximately 30 % in Iranian adults by the ATP III definition [38, 39]. It is likely that using different population criteria, including different age categories, gender ratios, living areas and physical activity levels, might have influenced the reported prevalence of MetS among studies in Iran. Further, the patterns of MetS prevalence in other countries, using different definitions of MetS, have also reported similar variations. An earlier study among Australians reported that one in three was identified with MetS by the IDF definition, while one in five was identified with MstS by using the WHO and ATPIII definitions and it was slightly less when the EGIR definition was used [40].

Previous studies have shown that individuals with MetS were at higher risk of CVD development in the near future (approximately 10 years) even after considering the confounding effects of other major risk factors such as age, sex, smoking, and hypercholesterolemia [41–46] which is in line with our results. Although the current study found that the WHO and IDF criteria for MetS was related to a high risk for CVD events (HR: 1.92 and 1.65 respectively), their requirements (insulin resistance or abdominal obesity) make it difficult to diagnose high risk individuals without insulin resistance or abdominal obesity. There was also a slight difference in CVD risk between the JIS, AHA-NHBI and ATP III definitions, which was principally because of the variation in their definition and threshold for impaired fasting glucose and WC. The definition which has higher sensitivity and identified a higher number of individuals who are at risk of CVD is the best. In the current study, the AHA-NHBI definition was associated with higher prevalence of MetS, as well as higher CVD risk. Thus, AHA-NHBI definition can be nominated as the best indicator to identify MetS for this study population. On the other hand, as the only difference between the AHA and JIS definitions is the WC threshold, our results showed that the suggested cut-off point for WC in the Iranian population may not be satisfactory and there is a need for redefining the WC cut-off point for the best estimation of CVD risk in this population.

The present study also found a significant association between the risk of CVD and MetS components. The associated risk was higher for glucose intolerance or diabetes (HR: 1.83, 95 % CI: 1.56–2.15) than any other MetS components, which is in line with previous research [47]. In addition, when the risk of CVD was examined based on the presence of MetS risk factors, more MetS components were associated with a higher risk of cardiovascular events. The risk of CVD for individuals having four and five components of MetS was 2.98 and 6.06 respectively (data not shown). Thus, it may also be necessary to examine the number of components in MetS individuals to identify the individuals at higher risk of CVD. Nevertheless, other risk factors of MetS including family history of diseases, age, gender, smoking, LDL or total cholesterol levels should be considered for CVD risk factors [48].

This study has some limitations. First, insulin resistance was not assessed directly, but instead, oral glucose tolerance test was used to estimate insulin sensitivity. Nevertheless, it is accepted that this measurement can be linked with insulin resistance [49]. Further, cohort studies are inherently limited for loss-to-follow up participants. However, the characteristics of individuals did not differ to a great extent as a result of drop-outs. On the other hand, the strength of this study is that to the best of our knowledge, it is the first study that has looked at the HR of CVD events occurrence based on MetS components and different definitions of MetS in a longitudinal study, in urban and rural areas in Eastern Mediterranean countries. Further, this study draws attention to the importance of having a national cut-off point for WC for the Iranian population, which could diagnose the individuals at higher risk of CVD. The present study also emphasises the importance of individual components of MetS for prediction of CVD risk.

## Conclusion

In conclusion, this representative sample of Iranian adults revealed a varied prevalence of MetS when using difference definitions of MetS. Further, follow-up of participants for more than 10 years showed that CVD risk was significantly higher in MetS participants, irrespective of the used definitions, as well as in participants with any risk factors of MetS. Overall, the AHA-NHBI and JIS definitions were better indicators because they were able to capture more individuals with MetS who were not identified by the EGIR and WHO definitions and were also at higher risk of CVD. Finally, the findings of this study emphasise the need for using the best possible population specific indicators for identifying MetS individuals. In addition, there is an urgent need for the development and implementation of appropriate protective measures, including lifestyle modifications, to improve all the MetS components. Control of individual components to prevent cardiovascular events is also necessary.

## Abbreviations

Mets: Metabolic syndrome
CVD: Cardiovascular disease
ASCVD: Atherosclerotic cardiovascular disease
HDL: High density lipoprotein
ICS: Isfahan cohort study
ICRI: Isfahan Cardiovascular Research Institute
WHO: World Health Organization
FBG: Fasting blood glucose
TC: Total cholesterol
LDL: Low density lipoprotein
VLDL: Very low-density lipoprotein
WC: Waist Circumference
EGIR: European Group for study of Insulin Resistance
NCEP ATP III: National Cholesterol Education Program, Third Adult Treatment Panel
IDF: International Diabetes Foundation
AHA: American Heart Association
NHLBI: National Heart, Lung, and Blood Institute
IFG: Impaired glucose tolerance
JIS: Joint interim statement
AMI: Acute myocardial infarction
UA: Unstable angina
HR: Hazard Ratio
IGT: Impaired glucose tolerance
